# Capturing health and eating status through a nutritional perception screening questionnaire (NPSQ9) in a randomised internet-based personalised nutrition intervention: the Food4Me study

**DOI:** 10.1186/s12966-017-0624-6

**Published:** 2017-12-11

**Authors:** Rodrigo San-Cristobal, Santiago Navas-Carretero, Carlos Celis-Morales, Katherine M. Livingstone, Barbara Stewart-Knox, Audrey Rankin, Anna L. Macready, Rosalind Fallaize, Clare B. O’Donovan, Hannah Forster, Clara Woolhead, Marianne C. Walsh, Christina P. Lambrinou, George Moschonis, Yannis Manios, Miroslaw Jarosz, Hannelore Daniel, Eileen R. Gibney, Lorraine Brennan, Thomas E. Gundersen, Christian A. Drevon, Mike Gibney, Cyril F. M. Marsaux, Wim H. M. Saris, Julie A. Lovegrove, Lynn J. Frewer, John C. Mathers, J. Alfredo Martinez, on behalf of the Food4Me Study

**Affiliations:** 1 Centre for Nutrition Research, Department of Nutrition, Food Science and Physiology, University of Navarra, C/Irunlarrea, 1, 31008 Pamplona, Spain; 2 CIBER Fisiopatología Obesidad y Nutrición (CIBERobn), Instituto de Salud Carlos III, 28023 Madrid, Spain; 3 Human Nutrition Research Centre, Institute of Cellular Medicine, Newcastle University, Newcastle Upon Tyne, NE1 7RU UK; 4 School of Psychology, University of Bradford, West Yorkshire, BD71DP UK; 5 Northern Ireland Centre for Food and Health, University of Ulster, Coleraine, BT52 1SA UK; 6 Hugh Sinclair Unit of Human Nutrition and Institute for Cardiovascular and Metabolic Research, University of Reading, Reading, RG6 6AA UK; 7 UCD Institute of Food and Health, UCD School of Agriculture and Food Science, University College Dublin, Belfield, Dublin, 4 Republic of Ireland; 8 Department of Nutrition and Dietetics, Harokopio University of Athens, 17671 Athens, Greece; 9 Institute of Food and Nutrition (IZZ), 02-903 Warsaw, Poland; 10 ZIEL Research Center of Nutrition and Food Sciences, Biochemistry Unit, Technische Universität München, 85354 Munich, Germany; 11 Vitas Ltd., Oslo Science Park, Gaustadalléen 21, 0349 Oslo, Norway; 12 Department of Nutrition, Institute of Basic Medical Sciences, Faculty of Medicine, University of Oslo, 0317 Oslo, Norway; 13 Department of Human Biology, NUTRIM School for Nutrition and Translational Research in Metabolism, Maastricht University Medical Centre, Maastricht, 6200 MD The Netherlands; 14 Food and Society Group, Newcastle University, Newcastle Upon Tyne, NE1 7RU UK; 15 Instituto de Investigaciones Sanitarias de Navarra (IDisNa), 31008 Pamplona, Spain; 16 Instituto Madrileño de Estudios Avanzados (IMDEA) Alimentacion, Madrid, Spain

**Keywords:** Food4Me, Personalised nutrition, Survey, Healthy eating index, Mediterranean diet score, NPSQ9, Nutritional status

## Abstract

**Background:**

National guidelines emphasize healthy eating to promote wellbeing and prevention of non-communicable diseases. The perceived healthiness of food is determined by many factors affecting food intake. A positive perception of healthy eating has been shown to be associated with greater diet quality. Internet-based methodologies allow contact with large populations. Our present study aims to design and evaluate a short nutritional perception questionnaire, to be used as a screening tool for assessing nutritional status, and to predict an optimal level of personalisation in nutritional advice delivered via the Internet.

**Methods:**

Data from all participants who were screened and then enrolled into the Food4Me proof-of-principle study (*n* = 2369) were used to determine the optimal items for inclusion in a novel screening tool, the Nutritional Perception Screening Questionnaire-9 (NPSQ9). Exploratory and confirmatory factor analyses were performed on anthropometric and biochemical data and on dietary indices acquired from participants who had completed the Food4Me dietary intervention (*n* = 1153). Baseline and intervention data were analysed using linear regression and linear mixed regression, respectively.

**Results:**

A final model with 9 NPSQ items was validated against the dietary intervention data. NPSQ9 scores were inversely associated with BMI (*β* = −0.181, *p* < 0.001) and waist circumference (*Β* = −0.155, *p* < 0.001), and positively associated with total carotenoids (*β* = 0.198, *p* < 0.001), omega-3 fatty acid index (*β* = 0.155, *p* < 0.001), Healthy Eating Index (HEI) (*β* = 0.299, *p* < 0.001) and Mediterranean Diet Score (MDS) (*β* = 0. 279, *p* < 0.001). Findings from the longitudinal intervention study showed a greater reduction in BMI and improved dietary indices among participants with lower NPSQ9 scores.

**Conclusions:**

Healthy eating perceptions and dietary habits captured by the NPSQ9 score, based on nine questionnaire items, were associated with reduced body weight and improved diet quality. Likewise, participants with a lower score achieved greater health improvements than those with higher scores, in response to personalised advice, suggesting that NPSQ9 may be used for early evaluation of nutritional status and to tailor nutritional advice.

**Trial registration:**

NCT01530139.

**Electronic supplementary material:**

The online version of this article (doi:10.1186/s12966-017-0624-6) contains supplementary material, which is available to authorized users.

## Background

A number of national strategies and programs focus on improving lifestyle and dietary habits for the prevention of non-communicable chronic diseases [1], especially those related to weight management [2, 3]. However, the perceived benefit of consuming certain foods is influenced by multiple individual factors, which may alter eating habits and dietary patterns. Identification of these factors and dietary patterns is an important challenge for the promotion of well-being and public health [4, 5]. Identification of barriers to the consumption of healthy foods is imperative to the design of effective behaviour change interventions and policies [6]. Moreover, information on barriers to healthy eating will help identify food-related perceptions that have the potential to negatively impact on dietary choices [7].

Perception of food healthiness is determined by numerous factors such as conventional and unconventional beliefs [4, 6], as well as consciousness/knowledge of food composition [8]. Such perceptions may affect attitudes towards foods consumption, resulting in under- or over-eating and causing unhealthy changes in body weight [8]. Previous studies have indicated positive associations between perception of healthy food intake and diet quality [9–11]. A preceding study on dietary patterns in a Spanish cohort showed that participants who presented with “*prudent”* or *“healthy”* dietary patterns reported greater proportions of positive perceptions of healthy eating than those who exhibited a *“Western”* or *“compensatory”* dietary patterns [5]. Similarly, the perception of healthy eating has been presented by some authors as a plausible predictor of behavioural intentions regarding food choices [12]. Such evidence suggests that a maintained positive perception of healthy intake, alongside other perceived values, might contribute to the adoption of healthy habits and food choices, including a reduced energy intake where appropriate, during a dietary intervention with personalised nutritional advice [13, 14].

Development of tools to assess health status has played an important role in health behaviour research [15]. The relationship between wellbeing and healthy eating is well established, which is the reason why many psychological and public health studies have tried to develop questionnaires aiming to collect information on complex issues like eating behaviour related to the development of chronic diseases such as obesity, diabetes, and cardiovascular events [16, 17]. These questionnaires require the use of representative data and sufficient accuracy before being used as early detection tools.

The current development of worldwide internet-based communications has highlighted the need for short and applicable tools for the screening of large populations [18]. The use of Internet-based platforms allows contact with large numbers of individuals with a good cost effectiveness [19], however such questionnaires would need to be tested in different settings and with large and heterogeneous populations. The present study was a cross-sectional and longitudinal analysis intended to design and validate a short nutritional perception questionnaire. It is anticipated that this screening tool may be used by health professionals to assess perceptions of eating behaviour and health status with the aim of predicting the optimal level of personalisation in nutritional advice via the internet.

## Methods

### Study population and study design

**Fig. 1:**
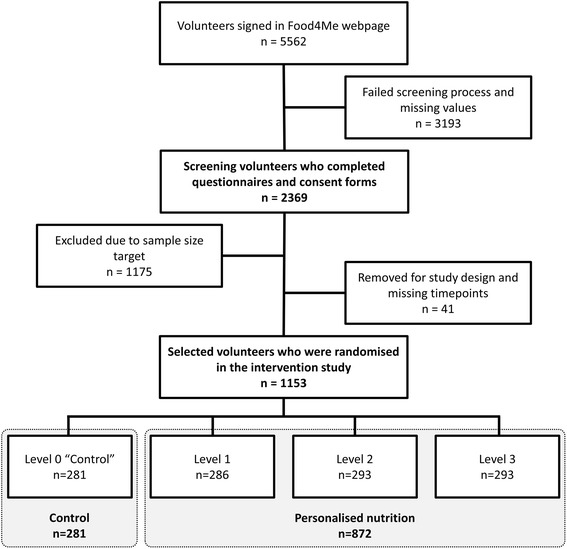
Flowchart for participant selection in the present study

The Food4Me study followed all required ethical standards, including the CONSORT guidelines (Additional file 1). Participants in the follow-up Nutritional Perception Screening Questionnaire-9 (NPSQ9) design were enrolled from the Food4Me study, which was a randomised controlled intervention trial designed to assess the effect of personalised nutrition advice on health-related behaviours across seven European countries [20]. Participants who signed up on the Food4Me webpage (http://www.food4me.org) and completed the initial screening processes were selected (*n* = 2369) for inclusion in the NPSQ9 design. These processes consisted of signing two informed consent forms if inclusion criteria for taking part in the Food4Me study were met [20], and providing information by answering the screening questionnaires (Table 1), on aspects regarding socio-demographics, medical history, lifestyle and dietary habits, health and eating self-perception, as well as responses to a validated Food Frequency Questionnaire (FFQ) [21, 22]. Volunteers selected for inclusion in the intervention and who completed the questionnaires at baseline and at 6 months (*n* = 1153), were used for the subsequent validation study and for association analyses with different dietary indices (Fig. 1). During the study, the volunteers were randomly assigned to one of four intervention groups receiving different types of personalised nutrition advice: Level 0 – control group – conventional non-personalised nutrition advice; Level 1 – personalised advice based on dietary data; Level 2 – personalised advice based on dietary and phenotypic data; and Level 3 – personalised advice based on dietary, phenotypic, and genotypic data. For the analysis of the effects of personalised nutrition advice, volunteers from Levels 1, 2, and 3, were pooled to evaluate the effects of personalising the nutritional advice, without taking into account the type of feedback provided.

**Table 1:** Characteristics of overall sample and by country

	*Overall*	*By country*							
		Germany	Greece	Ireland	Netherlands		Poland	Spain	United Kingdom
*n* (*n* of females)	2369 (1534)	343 (231)	262 (180)	238 (145)	398 (231)		253 (190)	634 (386)	241 (171)
Age (years)	40 ± 13	44 ± 14	38 ± 12	39 ± 13	48 ± 14		36 ± 13	38 ± 10	37 ± 13
Ethnicity									
Asian	11 (0.5%)	–	–	–	3 (0.8%)		–	–	8 (3.3%)
Black	2 (0.1%)	2 (0.6%)	–	–	–		–	–	–
Mixed	30 (1.3%)	5 (1.5%)	–	4 (1.7%)	5 (1.3%)		–	8 (1.3%)	8 (3.3%)
Chinese	1 (0.0%)	–	–	–	–		–	–	1 (0.4%)
White	2305 (97.3%)	332 (96.8%)	260 (99.2%)	234 (98.3%)	385 (96.7%)		253 (100.0%)	624 (98.4%)	217 (90.0%)
Other	20 (0.8%)	4 (1.2%)	2 (0.8%)	–	5 (1.3%)		–	2 (0.3%)	7 (2.9%)
BMI (kg/m^2^)	25.2 ± 4.7	24.3 ± 3.7	26.6 ± 5.8	25.4 ± 4.7	25.0 ± 4.2		24.6 ± 4.8	25.7 ± 4.8	24.9 ± 4.7
Weight status (by BMI)									
Under-weight	56 (2.4%)	8 (2.3%)	5 (1.9%)	8 (3.4%)	10 (2.5%)		11 (4.4%)	11 (1.7%)	3 (1.2%)
Normal weight	1247 (52.6%)	206 (60.1%)	115 (43.9%)	123 (51.7%)	214 (53.8%)		139 (54.9%)	308 (48.6%)	142 (58.9%)
Overweight	743 (30.8%)	97 (28.3%)	90 (34.4%)	65 (27.3%)	128 (32.2%)		68 (26.9%)	213 (33.6%)	71 (29.5%)
Obese	334 (14.1%)	32 (9.3%)	52 (19.9%)	42 (17.7%)	46 (11.6%)		35 (13.8%)	102 (16.1%)	25 (10.4%)
Energy intake reported (kcal)	2633 ± 775	2509 ± 678	2519 ± 744	2779 ± 772	2723 ± 760		2593 ± 779	2674 ± 816	2571 ± 805
Physical activity level (AU)	1.51 ± 0.10	1.50 ± 0.08	1.50 ± 0.11	1.53 ± 0.09	1.54 ± 0.10		1.50 ± 0.11	1.50 ± 0.10	1.54 ± 0.11
Smoke habit									
Non-smoker	1411 (59.6%)	201 (58.6%)	132 (50.4%)	164 (68.9%)	202	(50.8%)	197 (77.9%)	328 (51.7%)	187 (77.6%)
Ex-smoker	671 (28.3%)	112 (32.7%)	53 (20.2%)	57 (24.0%)	169	(42.5%)	38 (15.0%)	200 (31.6%)	42 (17.4%)
Current smoker	287 (12.1%)	30 (8.8%)	77 (29.4%)	17 (7.1%)	27	(6.8%)	18 (7.1%)	106 (16.7%)	12 (5.0%)

*BMI* Body Mass Index, *AU* Arbitrary Units

### Item selection

Data obtained from the questionnaire, specifically designed within the Food4Me study, and related to dietary habits, health perception, eating perception, and nutrition self-efficacy were used for the analyses. This questionnaire contained *Likert* scale questions related to *Nutrition Self-efficacy* [23], *Health locus of control* [24], *Self-report Habit Index* [25, 26], and *Dietary food choice/ habits* (Additional file 2: Table S1).

Socio-demographic questions, self-reported height and weight [27], the validated Food4Me FFQ [21, 22, 28], and biochemical values of dried blood spots [29], were analysed for associations with the scores obtained from the screening stage. These questionnaire items were coded and used to create a reduced aggregate score.

### Dietary assessment

For participants included in the randomization intervention study, diet quality indices were calculated at baseline (t0), 3 months (t3) and 6 months (t6), to assess the effect of personalised nutrition advice on dietary intake. Healthy Eating Index-2010 (HEI-2010) was calculated as described by Guenther et al. [30] according to consumption of food groups estimated using the FFQ. The Mediterranean Diet Score (MDS) was calculated using the PREDIMED 14-item screening tool [31]. Finally, Nutrient Adequacy Ratio (NAR), as described elsewhere [32], was estimated for the following nutrients: protein, carbohydrates, total fat, saturated fat, monounsaturated fatty acids (MUFA), polyunsaturated fatty acids (PUFA), omega-3 fatty acids, salt, fiber, calcium, iron, vitamin A, folate, thiamine, riboflavin, vitamin B_12_, and vitamin C, after the personalised nutrition intervention [33]. The NAR was specifically calculated for each nutrient, and the recommended intake values established in the intervention were used as the reference. Subsequently, the reported intake of each nutrient was computed as a percentage of the corresponding reference value, establishing levels of attainment. For some specific nutrients only under-consumption was considered inadequate, while for those nutrients in which excessive intake may also be considered inadequate, over-consumption was also taken into account. The Mean Adequacy Ratio (MAR) was a measure of overall diet adequacy including the mean of all the NAR components. For both NAR and MAR a score of 100% represented the ideal adequacy of intake reported, showing neither reduced nor excessive consumption.

### Chemical validation of intakes of fatty acids and of carotenoids using analysis of dried blood spots

Dietary fatty acid markers were determined via gas liquid chromatography combined with flame ionisation detection (GLC-FID) by Vitas Ltd. (www.vitas.no) as described previously [34]. Carotenoids were determined using high performance liquid chromatography with UV detection (HPLC-UV) on dried blood spots (DBS) cards with an appropriate stabilizer impregnated onto the DBS paper [35].

### Statistical analyses

To determine items for inclusion in the final NPSQ9 scale, the questions previously coded (Additional file 2: Table S1) were analysed through an exploratory factor analysis with the “least squares estimation” method and “varimax rotation”, to include the maximum amount of variance from the categorical variables.

Secondly, to test the suitability of the data used for the factor analysis, the Kaiser-Meyer-Olkin Criterion [36] and Bartlett’s test of sphericity [37] were performed. Scree plot and Eigen values higher than 1 were used for the selection of the factors to be included in the NPSQ9. By this method it was possible to collect the highest proportion of variance. For each factor, a step-wise selection (removing and rerunning the analysis) of the items was applied. Subsequently, the items presenting a factor loading greater than 0.3 for the model were selected and included in the aggregate score, which was calculated by summing the coded values of each question, thus providing a plausible range of scores from 0 to 30 (NPSQ9 score).

The internal reliability of the score items was evaluated by a Cronbach alpha analysis. Finally, a confirmatory factor analysis (CFA) was performed by means of structural equation modelling (SEM). To identify correlated uniqueness in the obtained factor model, modification of indices was checked, and goodness-of-fit indices were estimated. The resulting NPSQ9 score was used in the Food4Me participants who had been randomised to the personalised nutrition intervention (Fig. 1), in order to validate the results obtained with the exploratory analysis performed in the screening population. Model robustness was also tested by applying the model in different subgroups classified by sex and age (<45 years or ≥45 years) from the randomised volunteers.

A linear regression model adjusted for continuous variables (age, and physical activity) and categorical variables (sex, country, socio-economic status and smoking habits) was performed to test the association between the NPSQ9 score and anthropometrical characteristics, biochemical values, and diet quality indices (MDS and HEI) in the screening population. Furthermore, linear mixed regression models, adjusted also by age, sex, country, physical activity, socio-economic status and smoking habits, were used to analyse potential trends in variables categorised by tertiles of NSPQ9 score within the participants randomised in the Food4Me intervention. To analyse the effect of personalised nutrition advice during the intervention on the obtained NPSQ9 score, time-point and level of personalised advice interactions were included in the previously described mixed models for estimating the variation of each dependent variable on each tertile of the NPSQ9 score.

For descriptive analyses, differences between groups were assessed by chi-square for categorical variables, and by analysis of variance (ANOVA) adjusted for age, sex, country, physical activity, socio-economic status and smoking habits for continuous variables. All statistical analyses were performed using STATA statistical software (Stata IC version 12.0, StataCorp, College Station, TX, USA), and *p* values lower than 0.05 were considered significant.

## Results

Descriptive statistics among recruiting centres showed differences regarding population characteristics (Table 1). Exploratory factor analysis using an iterative process, carried out on the 2369 volunteers and including 22 questions from the screening questionnaire (Table S1, in Additional file 2), revealed a total of nine items with factor loadings higher than 0.3 after varimax rotation (Table 2). The Kaiser-Meyer-Olkin Criterion was 0.83 and Bartlett’s test of sphericity was highly significant (*p* < 0.001), indicating suitability of the results.

**Table 2:** Exploratory factor analysis for questionnaire item selection

	Factor loadings
*Factor 1: Management*	
*I Can Manage To Stick To Healthy Foods:*	
Even If I Need A Long Time To Develop The Necessary Routines	0.775
Even If I Have To Try Several Times Until It Works	0.819
Even If I Have To Rethink My Entire Way Of Nutrition	0.791
Even If I Do Not Receive A Great Deal Of Support From Others When Making My First Attempts	0.669
Even If I Have To Make A Detailed Plan	0.725
*Cronbach’s alpha =*	*0.875*
*Factor 2: Perception & Habits*	
Eating Healthily Is Something I Do Frequently	0.649
I Eat Healthily Without Having To Consciously Think About It	0.759
Eating Healthily Is Something I Don’t Have To Think About Doing	0.777
Do You Skip Meals And Replace Them With Snacks?	0.311
*Cronbach’s alpha =*	*0.732*

The nine items were aggregated into two groups (or factors) that were named “Management” and “Perception & Habits” respectively, to reflect the items included in each. The “Management” factor included items reflecting the self-reported capacity of the volunteers to select healthy foods, and the effort required to achieve healthy eating habits. The “Perception & Habits” factor included items related to the effort of selecting healthy foods, and one item involving substitution of meals with snacks. The correlation between the estimated factor scores and factors (factor determinacies coefficient) were higher than 0.978 for both factors. The analysis of internal consistency showed an acceptable Cronbach’s alpha of 0.792 for overall items, whereas the alpha values for each factor were 0.875 and 0.732, respectively (Table 2). Furthermore, results obtained in the exploratory analyses were confirmed by the CFA with the corresponding items (Figure S1, in Additional file 3). The goodness-of-fit values for the two factors model after the inclusion of four pairwise correlated errors showed acceptable ranges over the whole screening sample: RMSEA (0.037; 90% CI: 0.029–0.044), CFI (0.992). When the resulting model was applied to the sample of randomised volunteers, the results exhibited a satisfactory value for goodness of fit: RMSEA (0.031; 90% CI: 0.018–0.043), CFI (0.994). These results were also consistent when the model was carried out in categorised subsamples for sex and age: RMSEA (0.028; 90% CI: 0.012–0.040), CFI (0.994) and RMSEA (0.032; 90% CI: 0.019–0.044), CFI (0.992), respectively.

**Fig. 2:**
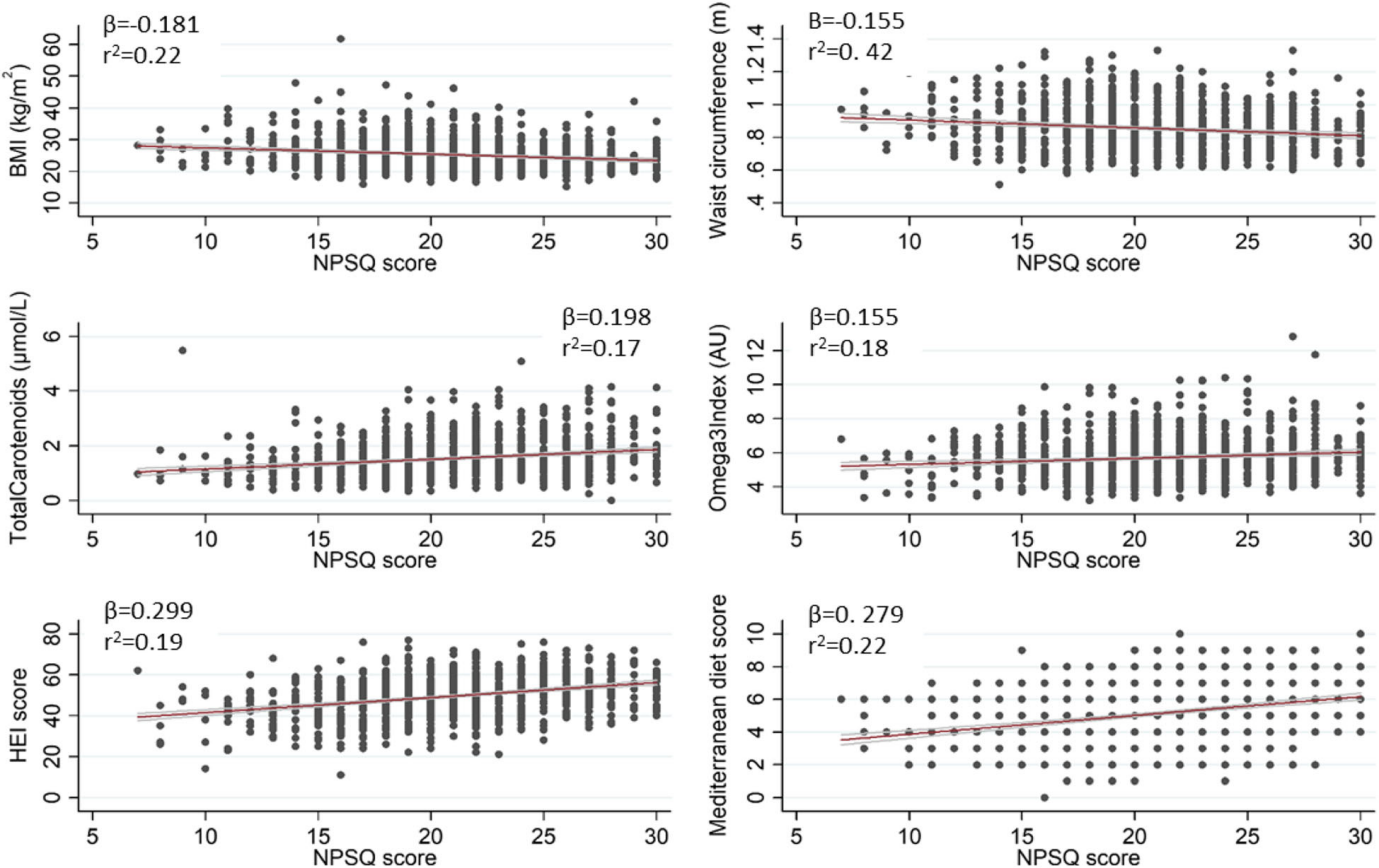
Association between Nutritional Perception Screening Questionnaire-9 (NPSQ9) Score with BMI, HEI score, total carotenoids in blood and Omega-3 fatty acid index in blood. All associations were highly significant (*p* < 0.001)

Differences in the screening sample characteristics were observed when volunteers were categorised into tertiles of NPSQ9 score (Table 3). Lower BMI values were observed in volunteers with a high NPSQ9 score, and physical activity level was lower for the volunteers in the first tertile. Regarding food consumption, the individuals ranked in the first tertile reported greater energy intake and higher intake of sweets & snacks, whereas there was an increased intake of cereal, egg, fruit, and vegetables in the upper tertiles. An association study within the randomised participants was carried out to evaluate previous results obtained at baseline on the anthropometrical, biochemical and diet quality indices (Fig. 2). Negative relationships were found for anthropometrical variables, showing *β*-values of −0.18 for BMI (*p* < 0.001) and −0.16 (*p* < 0.001) for waist circumference, whereas biochemical and dietary indices showed a positive association with *β*-values of 0.2 (*p* < 0.001) and 0.16 (*p* < 0.001) for total carotenoids and omega acid-3 fatty index, respectively, as well as 0.3 (*p* < 0.001) and 0.28 (*p* < 0.001) for HEI and MDS, respectively.

**Table 3:** Dietary characteristics of screening sample by Nutritional Perception Screening Questionnaire-9 (NPSQ9) tertiles

	*Tertile 1 (Low)* (Score 4–19)	*Tertile 2 (Medium)* (Score 20–23)	*Tertile 3 (High)* (Score 24–30)	*ρ* ^†^	*ρ* ^‡^
*n* (*n* of women)	934 (478)	805 (546)	630 (506)	**0.005** ^§^	
Age (years)	40 ± 12	41 ± 14	40 ± 13	0.069	0.408
Physical activity level (AU)	1.49 ± 0.10^a^	1.52 ± 0.10^b^	1.53 ± 0.10^b^	**<0.001**	**<0.001**
BMI (kg/m^2^)	26.4 ± 5.2^a^	25.0 ± 4.5^b^	23.9 ± 3.8^c^	**<0.001**	**<0.001**
Energy intake reported (kcal/day)	2723 ± 801^a^	2571 ± 733^b^	2577 ± 775^b^	**<0.001**	**<0.001**
Cereal (g/day)	42.9 ± 72.4^a^	58.4 ± 104.4^b^	64.9 ± 91.6^b^	**<0.001**	**<0.001**
Dairy products (g/day)	360.9 ± 254.6	374.2 ± 285.4	385.2 ± 284.3	0.540	0.303
Eggs (g/day)	32.3 ± 37.9^a^	30.9 ± 32.4^a^	37.5 ± 49.2^b^	**0.012**	**0.013**
Fats & Spreads (g/day)	21.2 ± 17.3	19.8 ± 14.9	20.3 ± 18.8	0.236	0.636
Fruit (g/day)	257.2 ± 237.6^a^	320.1 ± 248.6^b^	380.3 ± 301.4^c^	**<0.001**	**<0.001**
Meat & Fish (g/day)	201.9 ± 119.4	187.0 ± 116.2	199.6 ± 139.8	0.358	0.476
Soups & sauces (g/day)	94.7 ± 76.4	97.8 ± 79.3	97.6 ± 88.1	0.082	0.051
Sweets & snacks (g/day)	121.3 ± 93.9^a^	100.1 ± 83.1^b^	82.0 ± 69.7^c^	**<0.001**	**<0.001**
Vegetables (g/day)	188.1 ± 117.4^a^	229.1 ± 163.6^b^	282.6 ± 186.5^c^	**<0.001**	**<0.001**

*BMI* Body Mass Index, *AU* Arbitrary Units. ^†^ANOVA for least squared values adjusted by age, sex, country, smoking habits, and physical activity with Bonferroni post-hoc expressed by superscript letters; differences in letters show differences between groups with *p*-value < 0.05. ^§^ *p*-value for Chi-square test of distribution. ^‡^ *p*-value for linear trend

The trends during the intervention study (Table 4) showed significant reduction in BMI, waist circumference, plasma concentrations of glucose, cholesterol, total carotenoids, and MAR, whereas omega-3 fatty acid index, HEI and MDS were enhanced during the intervention. Differences in trends between tertiles 1 and 2 were observed in waist circumference and plasma glucose, whereas total carotenoids showed significant differences between tertiles 1 and 3. Furthermore, HEI and MAR exhibited differences in trends between the higher and lower tertile (Table 4). Despite the differences in trends during the intervention, the participants in tertile 3 maintained lower BMI and waist circumference, and higher levels of carotenoids and of omega-3 fatty acid index in blood, along with higher scores for HEI and MDS.

**Table 4:** Linear trend prediction through follow-up (0, 3 and 6 months) for changes by NPSQ9 tertiles of randomised volunteers

	Tertile 1 (Low)	Tertile 2 (Medium)	Tertile 3 (High)	*ρ* ^†^	*ρ* ^‡^
Score	7–19	20–23	24–30	–	–
*n* (women)	443 (258)	402 (237)	308 (174)	0.799^§^	–
BMI (kg/m^2^)	−0.16 ± 0.02^***^	−0.15 ± 0.02^***^	−0.12 ± 0.02^***^	0.934	0.340
Waist circumference (m)	−0.004 ± 0.001^***^	−0.007 ± 0.001^***^	−0.006 ± 0.001^***^	**0.024**	0.197
Glucose (mmol/L)	−0.10 ± 0.02^***^	−0.16 ± 0.02^***^	−0.12 ± 0.02^***^	**0.046**	0.472
Total colesterol (mmol/L)	−0.08 ± 0.02^***^	−0.09 ± 0.02^***^	−0.05 ± 0.02^*^	0.658	0.351
Total carotenoids (μmol/L)	−0.01 ± 0.01	−0.02 ± 0.01^*^	−0.05 ± 0.02^**^	0.257	**0.030**
Omega3 index (AU)	0.09 ± 0.02^***^	0.12 ± 0.02^***^	0.10 ± 0.03^***^	0.289	0.713
HEI score (AU)	1.75 ± 0.18^***^	1.13 ± 0.16^***^	1.05 ± 0.19^***^	**0.012**	**0.008**
MDS (AU)	0.21 ± 0.03^***^	0.15 ± 0.03^***^	0.12 ± 0.04^**^	0.201	0.070
MAR (%)	−1.84 ± 0.18^***^	−1.10 ± 0.16^***^	−1.12 ± 0.18^***^	**0.003**	**0.006**

*BMI* Body Mass Index, *AU* Arbitrary Units, *HEI* Healthy Eating Index, *MDS* Mediterranean Diet Score, *MAR* Mean Adequacy Ratio. *p*-values for linear trend represented by ^*^ for *p*-value <0.05; ^**^ for *p*-value < 0.01; ^***^ for *p*-value < 0.001

^†^ *p*-value for contrast of linear trend between Tertile1 and Tertile2; ^‡^ *p*-value for contrast of linear trend between Tertile1 and Tertile3; ^§^ *p*-value for Chi-square test of distribution

**Fig. 3:**
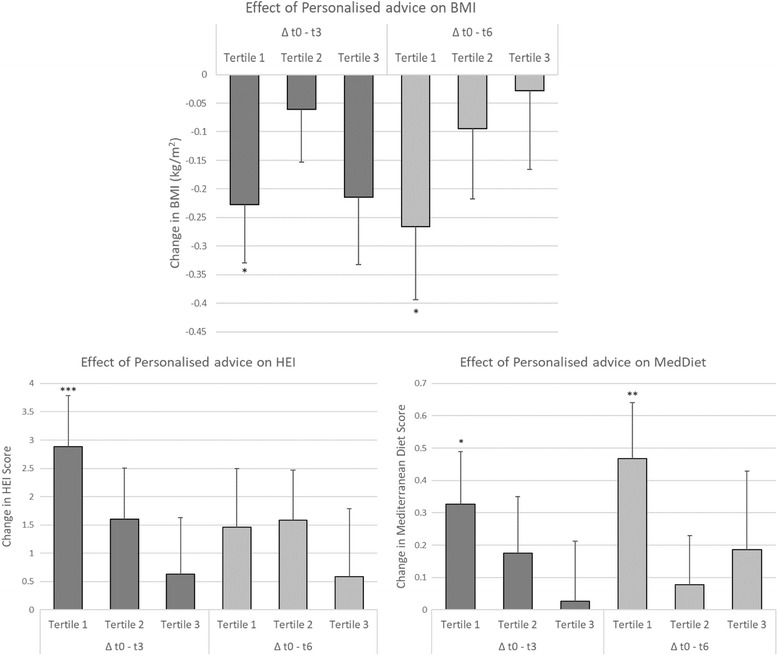
Effect of Personalised nutrition advice on each tertile of Nutritional Perception Screening Questionnaire-9 (NPSQ9) Score on the predicted change on BMI, HEI and Mediterranean diet score. Effects expressed in adjusted means with standard errors. Estimated *p*-values comparing the effect of personalised advice at follow-ups by NPSQ9 tertile. * *p*-value < 0.05; ** *p*-value < 0.01; *** *p*-value < 0.005

The effect of personalised nutrition advice on anthropometrical and dietary quality is shown in Fig. 3. Interestingly, a significant reduction in BMI was found for participants with low NPSQ9 scores receiving personalised advice as compared to the control group (only receiving general advice) at t3 (Δt0-t3: *β* = −0.23, 95%CI = −0.43 to −0.03, *ρ* = 0.025) and t6 (Δt0-t6: *β* = −0.27, 95%CI = −0.52 to −0.02, *ρ* = 0.038). Furthermore, significant effects were observed for the diet quality indices: an increase for HEI at short-term, 3 months (Δt0-t3: *β* = 2.81, 95%CI = 1.06 to 4.66, *ρ* = 0.002), and also for MDS at both short and long-term, 6 months (Δt0-t3: *β* = 0.33, 95%CI = 0.01 to 0.65, *ρ* = 0.045; Δt0-t6: *β* = 0.47, 95%CI = 0.13 to 0.81, *ρ* = 0.007; respectively).

## Discussion

The main novelty of this study was the development of a screening tool based on health and eating status, through self-perception questions. Despite the numerous questionnaires developed in the last years, the combination of awareness items with the capacity of predicting health and dietary outcomes has not been properly addressed so far [38–40].

This tool used items from the Food4Me screening questionnaire, which was validated to collect information relating to personalised nutrition [13]. Similarly, previous studies have sought to capture the information collected by validated questionnaires through reduced factor structure providing new reliable scales [40, 41] or validating this new factor structure in other populations or subsamples [42–44].

Indeed, a previous study using the Spanish screening cohort of the Food4Me study has indicated that some of the items present in the questionnaires were related to specific dietary patterns [5]. In that study, significant ifferences were observed in the perceptions of healthy eating habits between participants who were characterized by “*Western*” and “*Compensatory*” dietary patterns compared to participants reporting “*Prudent*” and “*Healthy*” dietary patterns. Differences were also found in habits that have previously been found to be related to the development of obesity, such as the frequency of eating fried foods, or the frequency of skipping meals with snacks [45–47], which form part of the NPSQ9’s “Perception & habits” factor.

Our findings support the usefulness of emerging statistical tools, such as factor structure analysis and criterion validity, to reduce the number of questions related to perceptions of healthy eating habits. The questionnaires used for the development of the present screening tool were selected and adapted for the Food4Me study to evaluate the psychological determinants of acceptance of personalised nutrition [13], self-reported dietary intake [21, 22, 28], and self-reported anthropometrical measurements [27]. In this context, these statistical tools have been used to analyse the dimensions of new questionnaires [41, 48], and for validation in other populations [40, 49]. However, the use of these tools also enables the reduction of dimensions within questionnaires, accounting for the maximum variance in the lowest number of factors [25, 40]. Previous studies have validated shortened questionnaires by relating responses to eating behaviour [19], emphasising the importance of perceptions of healthy eating and providing valuable tools to screen large populations [18, 50]. Furthermore, one of the most common limitations of questionnaires developed Ad Hoc is the uncertainty of reproducibility, and it is important that the new screening tool is reproducible when used across different population groups. For these reasons, we tested the robustness of the model in different subgroups in our own population.

Regarding the selection of questions to be included in the NPSQ9, some comments are needed, as during the factor analysis and selection work, some potentially controversial issues arose. Regarding Factor 2, two apparently similar questions were included: “I Eat Healthily Without Having to Consciously Think About It” and “Eating Healthily is Something I Don’t Have To Think About Doing”. However, the correlation was not strong between them, which may be explained through the analysis of acquired habits and habit acquisition [26]. In this sense, “Conscious thinking” would refer to an acquired habit, where active intention is not involved, while in the second question, the “thinking of doing” implies an active intention from the subjects’ side on changing or acquiring a new habit [26], assuming that the participant’s intention, when registering in the Food4Me study, was to improve health through dietary change.

Regarding another question included in the Factor 2 group (“Healthy Factor”), related to Meal skipping and snacking (“Do You Skip Meals and Replace Them With Snacks?”), it must be noted that meal skipping and replacing meals by snacks is not a healthy behaviour. Indeed, not doing these actions is associated with healthier dietary habits, and relates to energy balance and micronutrient adequacy [51, 52]. Thus this item was included but with the score inversely coded, giving the highest score in this item to those subjects who never or almost never skip meals.

In the present research, an association was found between high NPSQ9 scores and anthropometric measurements, biochemical values and diet quality indices, in line with previous information [41, 53, 54]. Other studies also reported a relationship between body weight and perceptions related to appetite [49]. Some authors found associations between body weight and behavioural questionnaires linked with the presence of specific gene variants related to appetite regulation in adults [55] as well as children [56]. Heritability of satiety and responsiveness to food suggest that genetics may influence some aspects related to eating behaviours and may also alter metabolic pathways [57].

A possible reason for the relationship between NPSQ9 score and healthy body weight could be that individuals with better scores showed more frequent consumption of fish, vegetables and fruit as observed in the analysis of MDS components (Additional file 2: Table S2). Reported intake of fish was associated with higher NPSQ9 scores, and the results were validated by the omega-3 fatty acid index in blood [58, 59].

Fruit and vegetable consumption was confirmed by the measure of total carotenoid concentration in blood at baseline [60, 61]. Preceding studies have shown that people with healthy eating perceptions show increased consumption of vegetables and fruit and higher diet quality indices, independently of socio-economic status, suggesting that healthy perception is representative of good nutrition [9]. Estimation of fruit and vegetable intake by short questionnaires has been widely studied by numerous researchers [62–64], also using the telephone [54, 65] or Internet [66]. In the present study, we used on-line contact, an approach in which the possibility of reaching large populations to promote healthier behaviours is notably increased, given the feasibility of using the internet worldwide [67], and the benefits and reliability of this approach [27, 29].

Analysis of the results from the intervention study showed high improvement in HEI for participants with low NPSQ9 scores. These individuals with a good perception of healthy eating showed greater capacity for, and willingness to, improve their diet [68]. Our results suggest that a score of 20 or less may be used as a cut-off to identify individuals with high risk of nutritional imbalance, although further analysis would be required. Results from the Food4Me study [64–66] demonstrated that personalised nutritional advice, based on self-reported information, led to improvement in participants’ dietary quality indices [69–71].

In the current investigation, participants’ reported intakes of fish, fruit and vegetables were validated by the biochemical measurements of omega-3 fatty acid index and total carotenoids in blood. The main limitation of the present work is the absence of repeated measures for the screening questionnaire, which would have allowed us to carry out a test/ re-test analysis to ensure repeatability of the results amongst the participants. Further research in this knowledge area is still needed, in order to demonstrate the efficacy and reproducibility of NPSQ9 as a screening tool and to determine robust cut-off values. Furthermore, it will also be necessary to determine whether online nutritional advice achieves dietary changes that are sustainable in the long-term.

## Conclusions

The aggregated score obtained from the NPSQ9 was associated with healthy body weight and diet quality, which could be used in health evaluation for early adaptation to healthy eating. Moreover, individuals with a low NPSQ9 score made greater improvements to their diet during the intervention with personalised nutritional advice provided on-line. Our results suggest that scores on the NPSQ9, with nine questionnaire items related to perception of healthy eating, could be used as a screening tool by dieticians and other health professionals to quickly estimate nutritional status and predict the appropriate level of personalisation in the nutritional advice.

## Additional files

Additional file 1: CONSORT CHECKLIST. (DOC 217 kb)

Additional file 2: Table S1. Set of all the questions included in the Item selection analysis. **Table S2.** Analysis of differences in MDS components by tertiles of NPSQ9 at baseline. (DOCX 25 kb)

Additional file 3: **Figure S1.** Flow diagram of the confirmatory factor analysis of selected items of Nutritional Perception Screening Questionnaire (NPSQ9) in the randomised sample. (PPTX 188 kb)

## Abbreviations

ANOVA: Analysis of variance
BMI: Body Mass Index
CFA: Confirmatory factor analysis
CFI: Comparative Fit Index
FFQ: Food Frequency Questionnaire
HEI: Healthy Eating Index
MAR: Mean Adequacy Ratio
MDS: Mediterranean Diet Score
MUFA: Monounsaturated fatty acids
NAR: Nutrient Adequacy Ratio
NPSQ: Nutritional Perception Screening Questionnaire
PUFA: Polyunsaturated fatty acids
RMSEA: Root Mean Square of Approximation
SEM: Structural equation modelling
