# Supplementary material for: Capturing health and eating status through a nutritional perception screening questionnaire (NPSQ9) in a randomised internet-based personalised nutrition intervention: the Food4Me study
Source: Int J Behav Nutr Phys Act. 2017 Dec 11;14:168. doi: 10.1186/s12966-017-0624-6 (PMC5725967; doi:10.1186/s12966-017-0624-6)
Supplement: Supplementary file 2 — Set of all the questions included in the Item selection analysis. Table S2. Analysis of differences in MDS components by tertiles of NPSQ9 at baseline. (DOCX 25 kb) [file 12966_2017_624_MOESM2_ESM.docx]

Additional file 2

**Table S1. Questions included for item selection**

| *Habits* | | |
| --- | --- | --- |
|  | How Often Do You Eat Your Main Meal Away From Home? | 0 'Never or up to once per month'  1 'Two to three times per month'  2 'Once per week'  3 'Twice or more per week' |
|  | How Often Do You Prepare A Meal "From Scratch"? | 0 'Every day' 1 '4-6 times per week' 2 '1-3 times per week' 3 '(Almost) never' |
|  | How Many Hot Or Cooked Meals Do You Normally Eat Per Day? | 0 'Never or rarely eat hot or cooked meals' 1 'One' 2 'Two' 3 'Three or more' |
|  | What Factor Do You Consider The Most Relevant When Selecting A Recipe? | 0 'Preparation time' 1 'Number of ingredients' 2 'Complexity' |
|  | How Much Time On Average Do You Spend Preparing A Main Meal? | 0 'Less than 10 minutes' 1 '10-20 minutes' 2 '20-30 minutes' 3 'Up to an hour' 4 'Over an hour' |
|  | Do You Skip Meals And Replace Them With Snacks? | 0 'Every day' 1 '4-6 times per week' 2 '1-3 times per week' 3 '(almost) never' |
|  | In The Past Month, How Often Did You Eat Fried Food? | 0 'One per day' 1 '5-6 per day' 2'2-4 per week' 3 'One a week' 4 '1-3 per month' 5 'Never' |
| *Health perception – Health Locus of Control* | | |
|  | I Can Be As Healthy As I Want To Be | 0 'Completely disagree' 1 'Disagree' 2 'Neither disagree nor agree' 3 'Agree' 4 'Completely agree' |
|  | I Am In Control Of My Health |  |
|  | I Can Pretty Much Stay Healthy By Taking Care Of Myself |  |
|  | Efforts To Improve Your Health Are A Waste Of Time |  |
|  | I Am Bored By All The Attention That Is Paid To Health And Disease Prevention |  |
|  | What's The Use Of Concerning Yourself About Your Health - You'll Only Worry Yourself To Death |  |
| *Eating perception – Self-Report Habits Index* | |  |
|  | Eating Healthily Is Something I Do Frequently | 0 'Completely disagree' 1 'Disagree' 2 'Neither disagree nor agree' 3 'Agree' 4 'Completely agree' |
|  | I Eat Healthily Without Having To Consciously Think About It |  |
|  | I Feel Weird If I Don't Eat Healthily |  |
|  | Eating Healthily Is Something I Don't Have To Think About Doing |  |
| *Healthy eating management – Nutrition Self-Efficacy* | | |
| I Can Manage To Stick To Healthful Foods: | |  |
|  | Even If I Need A Long Time To Develop The Necessary Routines | 0 'Very uncertain' 1 'Rather uncertain' 2 'Rather certain' 3 'Very certain' |
|  | Even If I Have To Try Several Times Until It Works |  |
|  | Even If I Have To Rethink My Entire Way Of Nutrition |  |
|  | Even If I Do Not Receive A Great Deal Of Support From Others When Making My First Attempts |  |
|  | Even If I Have To Make A Detailed Plan |  |
|  |  |  |

**Table S2: Analysis of differences in Mediterranean diet score components by tertiles of NPSQ9 at baseline.**

|  | Tertile 1 | |  | Tertile 2 | |  | Tertile 3 | |  | *ρ†* |  | *ρ‡* |
| --- | --- | --- | --- | --- | --- | --- | --- | --- | --- | --- | --- | --- |
| Olive oil ratio | 0.50 | ± 0.50 | ^a^ | 0.54 | ± 0.50 | ^a^ | 0.64 | ± 0.48 | ^b^ | **0.010** |  | **0.007** |
| Olive oil intake | 0.00 | ± 0.07 |  | 0.01 | ± 0.09 |  | 0.02 | ± 0.13 |  | 0.197 |  | 0.072 |
| Vegetables | 0.48 | ± 0.50 | ^a^ | 0.60 | ± 0.49 | ^b^ | 0.75 | ± 0.43 | ^c^ | **<0.001** |  | **<0.001** |
| Fruit | 0.49 | ± 0.50 | ^a^ | 0.65 | ± 0.48 | ^b^ | 0.72 | ± 0.45 | ^b^ | **<0.001** |  | **<0.001** |
| Processed meat | 0.84 | ± 0.36 | ^a^ | 0.92 | ± 0.28 | ^b^ | 0.92 | ± 0.28 | ^b^ | **0.010** |  | **0.015** |
| Fat spreads | 0.35 | ± 0.48 |  | 0.36 | ± 0.48 |  | 0.39 | ± 0.49 |  | 0.293 |  | 0.117 |
| Carbonated drinks | 0.98 | ± 0.15 |  | 0.99 | ± 0.12 |  | 0.99 | ± 0.10 |  | 0.693 |  | 0.392 |
| Wine | 0.07 | ± 0.25 |  | 0.09 | ± 0.29 |  | 0.08 | ± 0.28 |  | 0.725 |  | 0.829 |
| Legumes | 0.11 | ± 0.31 | ^a^ | 0.15 | ± 0.36 | ^a,b^ | 0.17 | ± 0.38 | ^b^ | **0.019** |  | **0.009** |
| Fish | 0.25 | ± 0.43 | ^a^ | 0.31 | ± 0.46 | ^a,b^ | 0.38 | ± 0.49 | ^b^ | **0.001** |  | **<0.001** |
| Sweets and pastries | 0.10 | ± 0.31 | ^a^ | 0.15 | ± 0.36 | ^b^ | 0.23 | ± 0.42 | ^c^ | **<0.001** |  | **<0.001** |
| Nuts | 0.11 | ± 0.31 | ^a^ | 0.16 | ± 0.36 | ^a,b^ | 0.20 | ± 0.40 | ^b^ | **0.005** |  | **0.001** |
| White meat | 0.28 | ± 0.45 | ^a,b^ | 0.22 | ± 0.42 | ^a^ | 0.33 | ± 0.47 | ^b^ | **0.004** |  | **0.037** |
| Tomato sauce | 0.01 | ± 0.11 |  | 0.01 | ± 0.12 |  | 0.01 | ± 0.08 |  | 0.534 |  | 0.491 |
|  |  |  |  |  |  |  |  |  |  |  |  |  |
|  | | | | | | | | | | | | |

†ANOVA analysis for least squared values adjusted by age, gender, country, smoke habit, physical activity with Bonferroni post-hoc expressed by superscript letters; differences in letters show differences between groups with p-value <0.05.
‡ p-value for linear trend
