# Supplementary material for: Capturing health and eating status through a nutritional perception screening questionnaire (NPSQ9) in a randomised internet-based personalised nutrition intervention: the Food4Me study
Source: Int J Behav Nutr Phys Act. 2017 Dec 11;14:168. doi: 10.1186/s12966-017-0624-6 (PMC5725967; doi:10.1186/s12966-017-0624-6)
Supplement: Supplementary file 3 — Figure S1. Flow diagram of the confirmatory factor analysis of selected items of Nutritional Perception Screening Questionnaire (NPSQ9) in the randomised sample. (PPTX 188 kb) [file 12966_2017_624_MOESM3_ESM.pptx]

## Slide 1
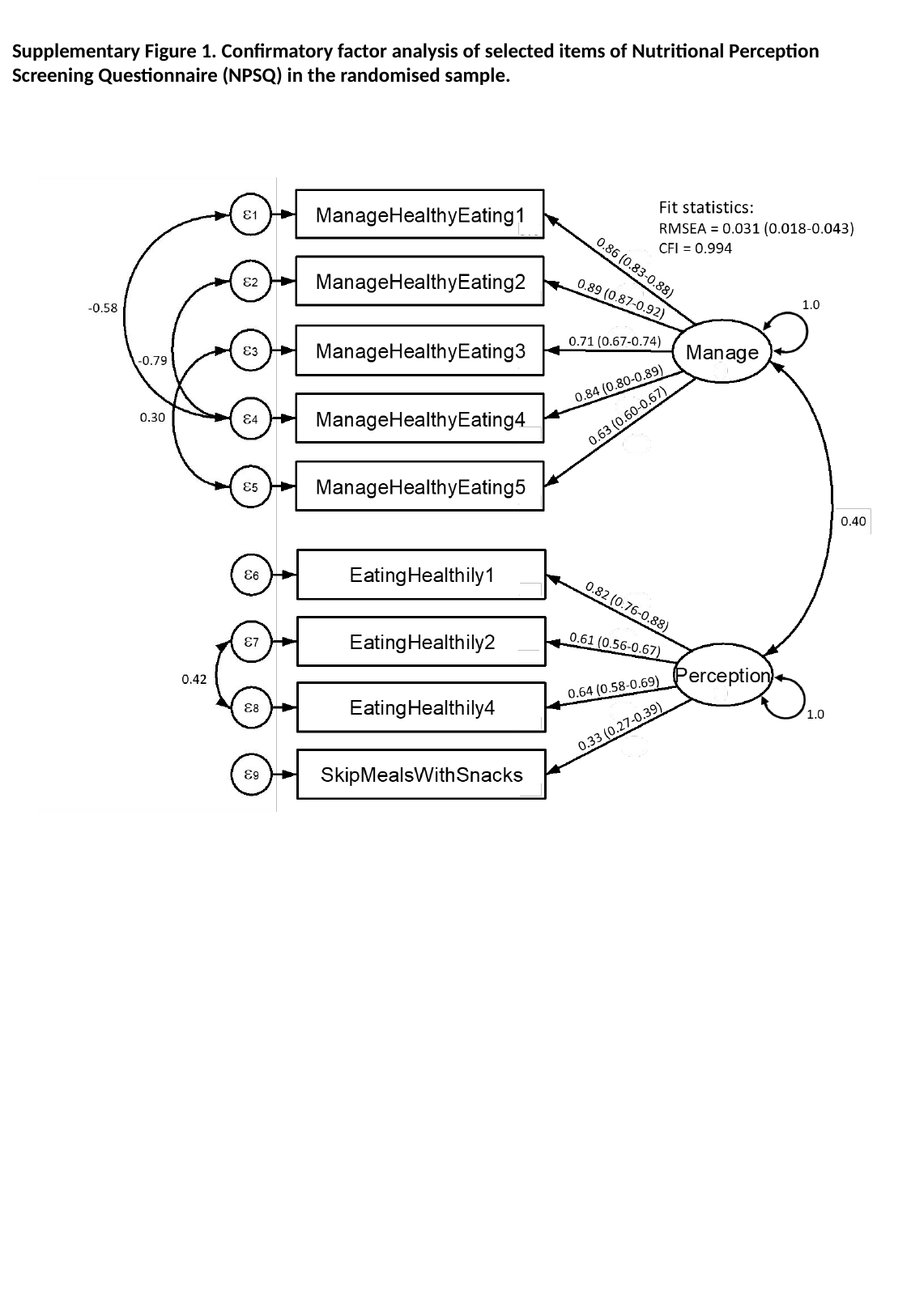

Supplementary Figure 1. Confirmatory factor analysis of selected items of Nutritional Perception Screening Questionnaire (NPSQ) in the randomised sample.
